# Alterations of selected iron management parameters and activity in food-restricted female Wistar rats (animal anorexia models)

**DOI:** 10.1007/s40519-013-0078-z

**Published:** 2013-10-18

**Authors:** Rafal W. Wojciak

**Affiliations:** 1 Department of Human Nutrition and Hygiene, Poznan University of Life Sciences, 31. Wojska Polskiego Str., 60-624 Poznan, Poland; 2 Department of Clinical Psychology, Poznan University of Medical Sciences, 70. Bukowska Str., 60-854 Poznan, Poland

**Keywords:** Female rat, Ferritin, Iron management, Activity, Animal anorexia model

## Abstract

**Aims:**

The aim of this study was to assess the influence of food-restricted diets (anorexia models) on iron management and activity of rats.

**Materials and methods:**

48 rats were divided into 6 groups: 1 control (K) and 5 testing groups (K/2, GI, GII, GIII, GIV). K was fed ad libitum. K/2 received half the portion of the diet of K. The other groups received 100 % of the diet eaten by K, but with different models of food restriction: GI—1 day on, 1 day starvation; GII—2 days on, 2 days starvation; GIII—3 days on, 3 days starvation; and GIV—4 days on, 4 days starvation. As a result, all testing groups ate half of the diet consumed by the control group. The concentrations of iron in selected tissues, ferritin, and selected iron management parameters in blood were examined, as well as the animals’ activities associated with food craving.

**Results:**

The animal anorexia models used in this study had a significant influence on the blood concentrations of hemoglobin (*p* < 0.01), hematocrit (*p* < 0.05), RBC (*p* < 0.05), iron levels in liver (*p* < 0.05), kidney (*p* < 0.001), and heart (*p* < 0.05), the serum ferritin concentration (*p* < 0.001) and the rats activity (*p* < 0.001); whereas there was no influence on the other parameters. Generally, the statistically negative effects of starvation models on iron management parameters and activity of animals were observed. However, these effects were dependent on the model of anorexia more than on the quantity of food intake.

**Conclusions:**

The negative effect of food deprivation on iron deficiency and rat activities were observed in all groups; however, the strongest effect was noticed in those animals subject to chronic starvation. Acute deprivations caused the reduction of activity in the rats, however, chronic starvation caused an increase in the activity of the first phase of the experiment, followed by a decline in the subsequent phase. It is possible that stress and frustration as well as depression may be caused by insufficient food intake, and as a result, by iron deficiency in a diet similar to human anorexia. However, more animal/human comparison studies are necessary.

## Introduction

Minerals are those nutrients which are completely exogenous. Their concentrations in tissues are mostly dependant on their level in food, bioavailability and the form in which metals occur. A low intake of trace elements is responsible for many disorders and dysfunctions, from invisible changes in enzymes and hormone activity to osteoporosis and bone loss. One of the most common disorders which is dependent on metal deficiencies is iron deficiency anemia. Iron deficiency and iron deficiency anemia affect billions of people worldwide. Recent studies suggest that up to 50 % of the world’s population could be affected by low iron levels [1]. McClung et al. [1] as well as other authors [2, 3] have reported that iron deficiency and iron deficiency anemia affects about 20 % of premenopausal women. Women who tend to be slim or lighter due to different than normal nutritional habits (vegetarian, low calorie, poor, etc.), are 3 times more likely to suffer from iron deficiency than those women with normal diets [1, 4–6]. In previous research the low tissue iron as a result of low iron intake was often observed [4–8]. Women are at risk of iron deficiency not only because of insufficient intake of this metal, but also the greatest risk of iron deficiency anemia occurs through the combined effects of suboptimal iron consumption and menstrual bleeding, which negatively influences iron balance. Studies of human subjects suggest that iron deficiency, as well as iron deficiency anemia in children and infants, is responsible for an immaturity of cognitive functions and behavioral development [9, 10]. The deficiency of this metal has also been related to fatigue and poorer general health and emotional and cognitive function in adults too, especially in women [11–13], affecting their daily activities. The earliest studies reported that low hemoglobin is associated with postnatal symptoms such as low energy, faintness/dizziness, painful perineal sutures and tingling of fingers [14–17].

Sixty percent of women aged 17–44 years [18] and more than 80 % of girls aged 14–17 years [18] have declared that they have tried different methods of weight loss such as alternative diets, an increase of physical activity, reducing food consumption or free-will starvation. More than 47 % of girls who have tried slimming applied free-will starvation as a method of weight loss for time spans ranging from a few days a year to even one or two days a week [18, 19]. In unpublished data from the author, a significant negative correlation between the depression scale and ferritin serum levels was observed in women starved 2 days a week. The association between postpartum depression, depressive symptoms, and low iron levels has been widely commented on recently [15–17]. Low mood, fatigue and depressive symptoms have also been observed in anorexics, however, these symptoms are usually co-associated with eating disorders, while not as result of that [18, 20]. After iron deficiency, anemia can lead to significant reductions, or even the disappearance of menstrual bleeding in patients with anorexia and bulimia. Interestingly these patients usually exhibit excessive physical activity [19, 20] which does not confirm common data on the reduction of physical activity as a result of low mood and depression during iron deficiency anemia. There are no data on the influence of food restriction diets on iron management in rats, nor on the alteration of animal activity in the environment of food deprivation. However, 48-h food deprivation is usually used as a one of the factors to induce stress in animal models of depression [21, 22]. In such models, the reduction of rats’ activities, suppression of appetite and a decrease in body mass are observed. These facts suggest that food deprivation can influence the level of daily activity in rats, maybe because of iron deficiency.

Taking the above information together, the aim of this study was to assess the iron status in rats exposed to chronic and acute starvation in different models of food deprivation (animal anorexia model). Additionally, the activity of the animals associated with food searching was also observed in this experiment.

## Experimental procedure

### Animals

Forty-eight female Wistar rats aged 8 weeks and with an initial body mass of 199 ± 18 g were obtained from the Licensed Animal Capacity in Brwinow, Poland. The rats used in this experiment were housed in a thermostatically controlled room (22 °C ± 2) on a 12 h light/dark cycle (lights on at 8:00 am), humidity (55–65 %) with free access to distillated water (ad libitum). All the animals were kept in individual stainless steel cages with neutral-plastic enamel. Animal care and handling for the experimental study were approved by the Local Regulatory Committee for animal studies (Approval No. 37/05).

### Food-restricted model

The experimental procedure of food deprivation in animals has been partially described before as an anorexia model by Siegfried [23] and was modified in this study by excluding physical activity (groups with chronic starvation: K/2 and GI). The acute starvation in rats (groups: GII, GIII and GIV) was used as a way of inducing depression in animal models [21, 22], although for not as long as in the presented study. At the beginning of the study, after a 3-day adaptation period, the animals were divided into six groups: control (K) and five testing groups (K/2, GI, GII, GIII, GIV). The K was fed ad libitum. The diet intake of this group was controlled every day and provided the base used to calculate the value of the diet given to the rest of the animals. K/2 received half the portion of the diet of that received by K. The rest of the groups received 100 % of the diet eaten by K, but with different models of food restriction: GI—1 day on, 1 day starvation; GII—2 days on, 2 days starvation; GIII—3 days on, 3 days starvation; and GIV—4 days on, 4 days starvation. As a result, all testing groups ate half of the diet normally consumed by the control group. All groups of rats were fed a standard, certified commercial diet for rodents Labofeed B (Morawski, Kcynia, Poland) for 48 days (6 × 8 days). The content of the diet was analytically assessed and was as follows: protein (19.9 ± 0.3 %), carbohydrates (69.9 ± 0.4 %), fat (2.7 ± 0.05 %), dry mass (88.4 ± 0.1 %), minerals total (7.5 ± 0.2 %), zinc (16.6 ± 1.5 mg/100 g), copper (2.3 ± 0.1 mg/100 g), iron (32.5 ± 2.5 mg/100 g).

At the end of the experimental period all animals were anesthetized by thiopental (40 mg/kg b.m.) intra-peritoneal injection, then dissected to collect femoral bone, kidney, liver, brain. The tissue samples were stored at −78 °C. The blood samples were collected directly from the heart to the PP tubes (with or without heparin) at room temperature and centrifuged at 5,000 rpm for 10 min and the serum was stored frozen at −78 °C, the iron-dependent parameters in all of the blood samples were measured immediately.

### Animals’ activity assessment

The activity of randomly selected animals was observed every day of the experiment (2 animals from each group, different rats on different days). Every approach to the feeder in the first hour (between 8.00 and 9.00 am), after putting the feeders to the cages, was recorded as the activity associated with food craving. In the days with food deprivation, the empty feeders were put into the cages of fasting rats.

### Analytical procedures

After being defrosted, the tissue samples were wet mineralized by microwaves with 5 mL HNO_3_ (supra pure, 65 %, w/w, Merck, Germany). After mineralization, the samples were quantitatively transferred to PP vials using deionized water to give about 25 mL. The iron concentration in the mineralized tissues samples was determined by the atomic spectrometry method with a graphite furnace spectrometer (AAS-5, Zeiss, Jena, Germany). As a control for the iron measurement, control materials (pig kidney—CRM, Brussels; human serum—Randox, UK) were used. The aqueous standard for samples was Fe(NO_3_)_3_ (Merck, Germany).

The ferritin concentration in serum was measured by the sandwich-ELISA method using standard rat ferritin ELISA kit (BioVendor, EU).

Hb (hemoglobin), Hct (hematocrit), RBC (red blood cell), MCH (mean corpuscular hemoglobin), MCV (mean corpuscular volume), MCHC (mean corpuscular hemoglobin concentration) and TIBC (total iron binding capacity) in the blood were measured using standard analytical procedures certified by a commercial laboratory, “Diagnostyka”.

### Data analysis and statistic

Data were expressed as the arithmetic mean ± SD. Group differences were assessed using the paired *t* Student test. To measure the influence of the model of starvation on all analyzing parameters, one-way ANOVA followed by Fisher’s least significant differences was used. Data were deemed significant when *p* < 0.05.

## Results

The baseline data of rats used in the study are displayed in Table 1. At the beginning of the experiment all animals had a similar body weight (199 ± 19 g). Significantly, the daily food intake was twice as high in the control group (K) (18.8 g/day/rat) than in others (~9.5 g/day/rat). The food intake in rats, calculated on the days when the diet was served, did not differ between the control and starvation groups, and was twice as high as those of rats who ate half levels of the daily food rations. The food-restricted rats systematically lost weight when compared to their body weight at the beginning of the study (ca. −45 g; body weight, ca. 155 g). However, the body weight of the control group increased (+25.5 g; body weight, 220.4 g). The greatest body weight loss was noted in the group starved 3 days (−53 g), the lowest in K/2 (41.6 g). However, all the models of food deprivation used in this experiment had a significant effect on reducing the weight of animals’ organs and their proportion of the total body mass was similar or higher than in the control group.

**Table 1:** General developmental and nutritional characteristics of the groups of rats (mean ± SD for 8 rats)

Lp.	Parameter	Experimental groups						ANOVA (*F*, *p*)
		K	K/2	GI	GII	GIII	GIV	
1.	Diet intake (g/day/rat)	18.8 ± 1.6^b^	9.9 ± 0.9^a^	9.9 ± 10.0^a^	9.2 ± 9.5^a^	9.3 ± 9.9^a^	9.7 ± 9.8^a^	8.82, *p* < 0.001
2.	Diet intake during food serving days (g/day/rat)	18.8 ± 1.6^b^	9.9 ± 0.9^a^	19.7 ± 6.0^b^	18.5 ± 2.7^b^	19.5 ± 1.1^b^	19.4 ± 1.1^b^	30.2, *p* < 0.001
3.	Body weight gain (g/56 days)	25.5 ± 9.2^c^	−41.6 ± 12.3^b^	−49.0 ± 10.1^ab^	−43.3 ± 12.6^b^	−53.0 ± 6.6^a^	−44.0 ± 8.7^ab^	61.1, *p* < 0.001
4.	Body weight at the end of study (g)	220.4 ± 17.9^b^	155.1 ± 7.4^a^	149.2 ± 11.6^a^	156.1 ± 11.9^a^	148.1 ± 20.1^a^	158.2 ± 10.9^a^	27.4, *p* < 0.001
5.	Organ weights at the end of study (g)							
	Liver (% of the body)	5.09 ± 0.66 (2.31)^b^	3.79 ± 0.21 (2.44)	4.71 ± 0.81(3.16)^b^	4.64 ± 0.32 (2.97)^b^	4.78 ± 0.87 (3.23)^b^	5.16 ± 0.65 (3.26)^b^	4.1, *p* < 0.01
	Kidney (% of the body)	1.40 ± 0.11 (0.63)^c^	1.09 ± 0.04 (0.70)^a^	1.13 ± 0.09 (0.76)^ab^	1.22 ± 0.10 (0.78)^b^	1.11 ± 0.13 (0.75)^ab^	1.21 ± 0.11 (0.76)^b^	8.9, *p* < 0.001
	Heart (% of the body)	0.62 ± 0.05 (0.28)^c^	0.53 ± 0.04 (0.34)^a^	0.50 ± 0.03 (0.34)^a^	0.55 ± 0.05 (0.35)^ab^	0.52 ± 0.07 (0.35)^a^	0.59 ± 0.07 (0.37)^bc^	4.8, *p* < 0.01
	Spleen (% of the body)	0.40 ± 0.06 (0.18)	0.27 ± 0.04 (0.17)^a^	0.26 ± 0.04 (0.17)^a^	0.28 ± 0.04 (0.18)^ab^	0.25 ± 0.08 (0.17)^a^	0.33 ± 0.05(0.21)^b^	7.7, *p* < 0.001

^a,b,c^Significant differences at *p* < 0.05 between groups

Table 2 shows the iron concentration in tissues, iron management blood parameters and activity levels of the rats. The experimental models of food deprivation used in this study had a significant influence on blood concentrations of Hb (*p* < 0.01), Hct (*p* < 0.05), RBC (*p* < 0.05), iron levels in liver (*p* < 0.05), kidney (*p* < 0.001), heart (*p* < 0.05), serum ferritin concentration (*p* < 0.001) and the rats’ activity (*p* < 0.001); whereas there was no influence on the MCV, MCH, MCHC, TIBC, serum and spleen iron levels. Generally, the reductive effects of starvation on both iron management parameters and the activity of animals were observed, albeit with some exceptions. The mean levels of Hb (14.5 g/dL), Hct (44 %) and RBC (8.2 million/μL) in K/2, GI and GII were similar to those in K, whereas in GIII and GIV the levels of these parameters were statistically significantly lower than in K (Hb 13.5 g/dL, Hct 40.5 % and RBC 7.5 million/μL). The mean iron liver concentration in K (1.62 mg/g d.m.) was significantly lower than in K/2 (1.94 mg/g d.m.) and higher than in GIV (1.46 mg/g d.m.), whereas it was statistically similar to that in GI (1.91 mg/g d.m.), GII (1.77 mg/g d.m.) and GIII (1.58 mg/g d.m.). A similar situation was observed in the concentration of iron in heart tissue, where a significantly higher level of this metal was noticed in K/2 (474.5 μg/g d.m.) and a lower level in GII (345.9 μg/g d.m.) than in the control group (405.1 μg/g d.m.). There were no significant differences between mean levels of iron in the heart in K and GI (404.4 μg/g d.m.), GIII (451.1 μg/g d.m.), and GIV (412.5 μg/g d.m.). A strong accumulation of iron was observed in kidney tissues. All of the starvation groups had a significantly higher level of this metal than in the control group (321.1 μg/g d.m.), although the highest was observed in GIV (535.6 μg/g d.m.) which was significantly higher than in other groups (ca. 470 μg/g d.m.). The mean concentrations of serum ferritin were significantly lower in K/2, GI, and GII (1.54, 1.72 and 1.69 μg/dL, respectively) than in the control (2.82 μg/dL), whereas in GIII and GIV were significantly higher (3.29 and 5.26 μg/dL, respectively). Similar concentrations of MCV (ca. 53 fL), MCH (ca. 18 pg), MCHC (ca. 33 g/dL), TIBC (ca. 500 μg/dL), serum iron (ca. 250 μg/dL) and spleen iron (ca. 1.2 mg/g d.m.) were observed in all groups.

**Table 2:** Iron management parameters concentrations in tissues and the activity of rats ($\overline{x}$ ± SD)

Lp.	Parameter	Experimental groups ($\overline{x}$ ± SD)						ANOVA (*F*, *p*)
		K	K/2	GI	GII	GIII	GIV	
1.	Hb (g/dL)	14.64 ± 0.26^b^	14.85 ± 0.82^b^	14.76 ± 0.79^b^	14.36 ± 0.48^b^	13.48 ± 0.64^a^	13.50 ± 0.78^a^	4.7, *p* < 0.01
2.	Hct (%)	44.07 ± 1.22^b^	44.63 ± 3.33^b^	43.59 ± 3.20^b^	43.50 ± 1.94^b^	40.67 ± 2.06^b^	40.54 ± 1.95^a^	2.9, *p* < 0.05
3.	RBC (million/μL)	8.29 ± 0.40^b^	8.37 ± 0.43^b^	8.26 ± 0.62^b^	8.10 ± 0.36^b^	7.41 ± 0.56^a^	7.53 ± 0.41^a^	3.0, *p* < 0.05
4.	MCV (fL)	53.25 ± 1.49	53.30 ± 1.41	52.83 ± 1.43	53.78 ± 1.47	53.94 ± 1.70	53.89 ± 1.09	0.6, NS
5.	MCH (pg)	17.72 ± 0.85	17.79 ± 0.44	17.93 ± 0.51	17.78 ± 0.64	17.89 ± 0.59	17.95 ± 0.36	0.2, NS
6.	MCHC (g/dL)	33.23 ± 1.06	33.33 ± 1.07	33.90 ± 0.71	33.02 ± 0.93	33.17 ± 0.22	33.31 ± 0.74	0.8, NS
7.	TIBC (μg/dL)	502.0 ± 35.8	498.3 ± 68.2	512.3 ± 78.2	473.8 ± 68.2	495.9 ± 95.8	483.2 ± 49.8	2.1, NS
8.	Serum Fe (μg/dL)	230.6 ± 91.0	246.1 ± 90.5	244.0 ± 97.8	272.0 ± 69.2	263.5 ± 36.7	242.0 ± 40.6	0.2, NS
9.	Liver Fe (mg/g d.m.)	1.62 ± 0.37^b^	1.94 ± 0.26^c^	1.91 ± 0.27^bc^	1.77 ± 0.43^bc^	1.58 ± 0.27^ab^	1.46 ± 0.21^a^	2.7, *p* < 0.05
10.	Kidney Fe (μg/g d.m.)	321.1 ± 60.7^a^	485.7 ± 107.7^b^	487.7 ± 62.1^b^	466.1 ± 62.4^b^	476.5 ± 32.0^b^	535.6 ± 91.6^c^	6.6, *p* < 0.001
11.	Heart Fe (μg/g d.m.)	405.1 ± 161.7^b^	474.5 ± 81.9^c^	404.4 ± 50.8^ab^	345.9 ± 71.8^a^	451.1 ± 96.9^bc^	412.5 ± 99.0^bc^	2.8, *p* < 0.05
12.	Spleen Fe (μg/g d.m.)	1.097.3 ± 366.5	1.401.7 ± 341.3	1.182.7 ± 305.9	1.350.8 ± 664.1	1.445.1 ± 316.9	1.195.2 ± 313.2	0.6, NS
13.	Serum ferritin (μg/dL)	2.82 ± 0.27^c^	1.54 ± 0.17^a^	1.72 ± 0.13^b^	1.69 ± 0.18^b^	3.29 ± 1.23^d^	5.26 ± 2.16^e^	17.1, *p* < 0.001
14.	Rats activity (no. of approaches to the feeder)	3.60 ± 0.35^d^	3.17 ± 1.58^c^	2.98 ± 1.42^c^	2.44 ± 1.45^b^	1.35 ± 0.96^a^	1.41 ± 0.87^a^	30.3, *p* < 0.001

*NS* not significant

^a,b,c^Significant differences at *p* < 0.05 between groups

**Fig. 1:**
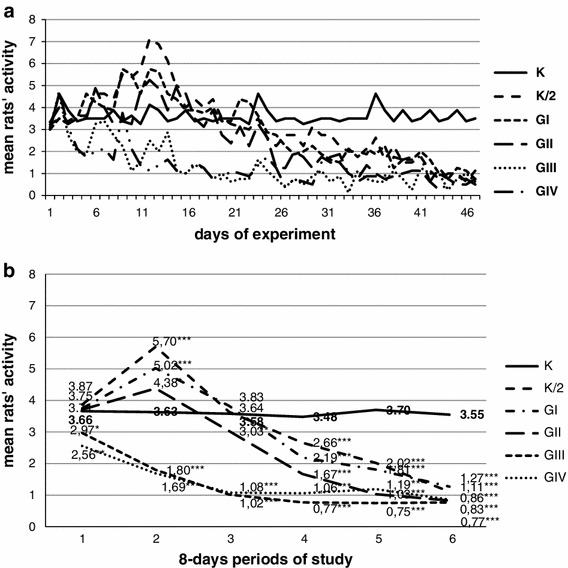
The mean activity of animals observed in all days of the experiment (**a**) and in after division into the 6 eight-day periods (**b**). *Asterisk* statistically significant differences between control and starvation groups at *p* < 0.05. *Double asterisk* statistically significant differences between control and starvation groups at *p* < 0.01. *Triple asterisk* statistically significant differences between control and starvation groups at *p* < 0.001

The rats’ activity, presented as the mean numbers of contacts with the feeder in the first hour after it was placed into the cages (from every day of the experiment period) was significantly lower in all starvation groups than in the control group (3.6). However, with the increasing number of days of starvation (from one to four), the mean activity of rats decreased, from ca. 3 in K/2 and GI—a significantly lower amount than in those in GII (2.4)—to the lowest in GII and GIV (ca. 1.4). In the detailed analyses of rats’ activity on all days (mean from all rats in groups, Fig. 1a), in the few first days all rats presented similar activity, whereas by about the sixth day, the activity of the groups exposed to starvation changed. The activity of rats exposed to starvation for 3 and 4 days (GIII and GIV) decreased rapidly, whereas the rest of the starvation groups (K/2, GI and GII) witnessed an intensification of the rats’ activity at the beginning of experiment, and then, after the 26th day, a reduction to the level of the acute starvation groups. This effect is easier to observe in Fig. 1b, where the activity is presented as the mean from all 6 eight-day periods. There were no statistical differences between the activity of control (3.7) and K/2 (3.9), GI (3.7), and GII (3.7), whereas significant hyperactivity in GIII (3.0, *p* < 0.05) and GIV (2.6, *p* < 0.01) was observed in the first period of the experiment. In the second period, greater differences in activity were noticed. However, the chronic starvation groups and GII demonstrated increased activity (from 4.4 at *p* < 0.05 in GII to 5.0 and 5.7 at *p* < 0.001 in GI and K/2, respectively), whereas activity in GIII and GIV decreased (1.8 and 1.7, at *p* < 0.001) in comparison to K (3.6). In the third period of the experiment, the activity of the chronic starvation groups started to decrease (the activity of the K/2 and GI reduced to the level of K; ca. 3.6, while the GII significantly decreased, 3.0 *p* < 0.05), whereas the acute starvation groups leveled off (the GIII and GIV, ca. 1.0). In the remaining periods, the activity of K/2, GI and GII decreased gradually to the level of the acute starvation groups. In the sixth and final period of the experiment, the activity of all starvation groups (K/2-1.11, GI-1.27, GII-0.83, GIII-0.86, and GIV-0.77) was significantly lower (*p* < 0.001) than that in the control group (3.6).

## Discussion

Although studies have shown that free-will starvations are regularly used, often as a simple method to lose weight, and especially by women of different ages [18–20], the scientific data on the influence of food deprivation on health are poor. Recently studies [4, 6] have shown that restricted diets, based on low-calorie products, can induce trace element deficiencies. Observations of groups of women with anorexic behavior [7], vegetarians [4] or slimming [6] indicate that such diets can significantly reduce zinc, magnesium, chromium, and iron levels. Iron deficiency and iron deficiency anemia are common disorders wholly dependent on iron intake from food. Therefore, it is often recognized in people who eat mono-nutrient or poor diets, for example those excluding meat or other products. Iron deficiency has been recognized in more than 50 % of women of reproductive age [17, 24]. However, the cause of these disorders lies not only in insufficient iron intake stemming from a poor diet, but also too heavy bleeding during menstrual periods [15, 16].

Furthermore, there are no data regarding the influence of food deprivation on iron levels in animal studies. In the presented experiment, different animal models of food deprivation, from chronic to acute, were applied to female Wistar rats and the iron status was measured. The chronic starvation did not change iron-dependent parameters, but acute starvation (3 and 4 days in every 8-day period) significantly decreased the Hb, Hct and RBC as well as iron concentrations in the liver. There were neither changes in MCV, MCH, MCHC, TIBC nor in iron levels in serum, heart and spleen, while in the kidney, the cumulative effect of all models of food restriction was observed. Although a significant reduction of iron parameters was observed strongly in acute starvation, this does not mean that iron deficiency was not observed in other models of food restriction. Many authors [11, 13, 16] are of the opinion that ferritin concentration in serum (or plasma) is strongly associated with iron storage in tissues, rather than iron levels in serum where the homeostasis processes maintain a constant level of iron. Proof of this can be seen in alterations of iron concentration in soft tissues (liver, kidney) and significantly lower levels of serum ferritin—about 50 % in starvation groups, in comparison to control groups. The liver is the main storage point of iron in an organism, hence the alterations of iron concentration in this tissue were observed in rats. There was an increase in chronic starvation group (K/2) and a strong reduction in the acute group (GIV). This data suggest that the changes in iron management are the result of both chronic and acute starvation.

Ferritin is a protein that plays an important role in the storage of iron in the body and the latest data suggest that it can be a multidimensional biomarker. Goswami et al. [11] have reported that this protein is now emerging as a very important factor in the pathogenesis of diseases such as atherosclerosis, cancer (especially liver, kidney and breast cancers), neuropsychiatric disorders (Alzheimer’s disease, Parkinson’s disease, ADHD, multiple sclerosis, etc.), and diabetes mellitus. The responsibility of this protein in pathology is probably strongly associated with a number of mechanisms in which ferritin functions, such as in pro-oxidant and pro-inflammatory pathways [11, 24, 25]. Numerous recent studies [2, 14, 16] have focused on low iron and ferritin levels and their association with low mood and depression. Steward and Hirani [13] are of the opinion that depressive symptoms are associated with low Hb concentrations as well as with low ferritin, as a result of iron deficiency. Other authors have come to similar conclusions [1, 2, 15, 26]. The association between low blood (serum or plasma) levels of ferritin with postpartum depression has been reported by many authors [15, 17]; however, some authors have not found such relationship [24].

There are no data on the association between iron status, ferritin concentrations and behavior in animals. However, different food deprivation models have been used to induce stress in animal models of anxiety disorders, research on stress, and depression [21, 22] as well as in anorexia animal models [23]. In this study, the significant influence of starvation on rats’ activity was observed. Interestingly, at the beginning of the experiment chronic starvation (K/2, GI, GII) induced hyperactivity, whereas acute starvation led to a reduction in activity. The phenomenon of hyperactivity may be associated with the sensation of hunger, which results in an active search for food. Herbert et al. [12] observed how 24-h food deprivation affects hunger levels and affective experiences in women. The significant effect of reduction of acute food deprivation on the rats’ activity was probably associated with the psychological concept of learned helplessness, discovered in an animal study by Seligman et al. [27] and observed in humans by Abramson et al. [28]. Rats were very quick to learn that after a prolonged period of food starvation the feeders became empty. The reduction of activity in all the starvation groups as the experiment progressed could not be explained by the concept of learned helplessness, however. Rather, it was associated with iron deficiency anemia and low levels of ferritin, which could cause depressive symptoms. Many authors have reported that iron deficiency and low concentration of ferritin cause different psychological symptoms such as fatigue, low mood, poorer general health and emotional and cognitive functioning as well as depression in women and children [1, 9, 10, 13]. The significant correlation effect between low ferritin concentration and hypoactivity in rats was observed also in this study.

On the other hand, in this study a higher than normal level of ferritin was observed in animals exposed to acute starvation. High serum ferritin concentrations in women with anorexia have already been reported by other authors [29–31], as has a return of this level to normal after nutritional rehabilitation [29, 30]. Kennedy et al. [29] suggest that anorexic patients generally have a low or normal hematocrit suggesting a reduction also in red blood cells. This process involves destruction of red blood cells and the iron released from this process is stored as ferritin, which is consistent with the elevated ferritin concentration. Some of authors have reported that the number of red blood cells decreases during malnutrition associated with anorexia nervosa [30, 32]. The significant decrease in the levels of red blood cells was also observed in acute starvation rats.

Iron deficiencies cannot always be compensated for with supplements. Recently published data from Powell et al. [33] has reported that dietary fortified iron intake is negatively associated with quality of life in patients, probably as a result of low bioavailability as well as an antagonistic mechanism with other metals. The toxic effect of high doses of iron is also known [17, 25].

Although symptoms of depression are often recognized in anorexia [34, 35] as well as iron deficiency anemia [30], there are no data combining both symptoms. It is possible that depression may be caused by insufficient food intake in anorexics, and as a result, by iron deficiency in the diet. It seems that further work on this topic is necessary and may contribute to a fuller understanding of the phenomenon of how iron deficiency can influence such delicate processes as mood, feelings, or emotions. Moreover, the animal studies may be helpful in explaining the impact of both how dietary restrictions can initiate the iron deficiency and also how food deprivation can change behavior by initiating hypoactivity through iron deficiency.
